# Multimorbidity patterns of and use of health services by Swedish 85-year-olds: an exploratory study

**DOI:** 10.1186/1471-2318-13-120

**Published:** 2013-11-06

**Authors:** Huan-Ji Dong, Ewa Wressle, Jan Marcusson

**Affiliations:** 1 Department of Clinical and Experimental Medicine, Division of Geriatrics, Faculty of Health Sciences, Linköping University, 581 85 Linköping, Sweden; 2 Department of Geriatric Medicine, County Council of Östergötland, Linköping, Sweden

**Keywords:** Multimorbidity, 85-year-old, Emergency-room visit, Hospitalization

## Abstract

**Background:**

As life expectancy continues to rise, more elderly are reaching advanced ages (≥80 years). The increasing prevalence of multimorbidity places additional demands on health-care resources for the elderly. Previous studies noted the impact of multimorbidity on the use of health services, but the effects of multimorbidity patterns on health-service use have not been well studied, especially for very old people. This study determines patterns of multimorbidity associated with emergency-room visits and hospitalization in an 85-year-old population.

**Methods:**

Health and living conditions were reported via postal questionnaire by 496 Linköping residents aged 85 years (189 men and 307 women). Diagnoses of morbidity were reviewed in patients’ case reports, and the local health-care register provided information on the use of health services. Hierarchical cluster analysis was applied to evaluate patterns of multimorbidity with gender stratification. Factors associated with emergency-room visits and hospitalization were analyzed using logistic regression models.

**Results:**

Cluster analyses revealed five clusters: vascular, cardiopulmonary, cardiac (only for men), somatic–mental (only for men), mental disease (only for women), and three other clusters related to aging (one for men and two for women). Heart failure in men (OR = 2.4, 95% CI = 1–5.7) and women (OR = 3, 95% CI = 1.3–6.9) as a single morbidity explained more variance than morbidity clusters in models of emergency-room visits. Men's cardiac cluster (OR = 1.6; 95% CI = 1–2.7) and women's cardiopulmonary cluster (OR = 1.7, 95% CI = 1.2–2.4) were significantly associated with hospitalization. The combination of the cardiopulmonary cluster with the men’s cardiac cluster (OR = 1.6, 95% CI = 1–2.4) and one of the women’s aging clusters (OR = 0.5, 95% CI = 0.3–0.8) showed interaction effects on hospitalization.

**Conclusion:**

In this 85-year-old population, patterns of cardiac and pulmonary conditions were better than a single morbidity in explaining hospitalization. Heart failure was superior to multimorbidity patterns in explaining emergency-room visits. A holistic approach to examining the patterns of multimorbidity and their relationships with the use of health services will contribute to both local health care policy and geriatric practice.

## Background

A growing number of studies have noted that an increasing number of chronic conditions is resulting in a substantial rise in the use of health service resources, and associated expenses will continue to rise [1-5]. Among the younger population, the predominant picture is that women report more chronic conditions and seek more health care than men [6,7]. In contrast, among the population of 85-year-olds, researchers found that women use the same or fewer health services than men [8,9]. However, these studies conducted no further analysis with regard to underlying factors in relation to the use of health services. In 2007, we started a population-based project on 85-year-old residents in Linköping municipality (Elderly in Linköping Screening Assessment, ELSA 85, Sweden). We studied morbidity and multimorbidity (at least two chronic diseases), living conditions, and visits to the general practitioner (GP) in relation to in-patient hospitalization [10]. Factors associated with in-patient care included an increased number of GP visits, more assistive technology, community assistance and multimorbidity [10]. Examining some of these factors, gender has been shown to influence several of the covariates; e.g., the use of assistive technology [11] and multimorbidity [12]. Moreover, studies on multimorbidity defined by a cut-off point did not reflect how the morbidities relate to each other. As reported by John et al. [13] and applied by Marengoni et al. [14] and Formiga et al. [15], some co-occurrences exceed a level expected by chance alone. Therefore, studies on multimorbidity may have to be explored in a more complex context; e.g., the effects of gender and clustering of diseases can be considered.

In Sweden, public resources are state controlled. Provisions for community services, assistive technology, and health care are funded by taxes and are universally available according to individual needs [16]. Individuals pay 150 SEK ($23) for a visit to a GP, 300 SEK ($46) to access emergency care and up to 80 SEK ($12) per day for a hospital stay [17]. With a GP referral to an emergency room (ER), a compensation payment of 150 SEK instead of 300 SEK is charged. The base of the health care system is primary care. Linköping, the largest town in Östergötland County, has a university hospital in which the primary care and the hospital disciplines have shared patient records via an electronic system (Cosmic) since 2007. A referral from a GP is mandatory for patients to visit a specialist whenever specialized health care is required. In practice, younger patients usually refer themselves to the ER whereas it is more common that older patients are referred after visiting their caregivers in primary care. The GP plays an important role in the further care of patients. We therefore consider consultations with a GP as a potential factor related to both ER visits and hospitalization.

The aim of the present study is to further examine the complexity of multimorbidity in relation to the use of health services. We conducted analyses with gender stratification to investigate morbidity patterns and their associations with ER visits and hospitalization. Nationwide, these two outcomes account for substantial spending in health care.

## Methods

### Sample

All eligible inhabitants were individuals born in 1922 and residing in Linköping municipality, Sweden (n = 650; 235 men and 415 women). The inclusion period for this study was one year (between March 2007 and March 2008). Postal questionnaires and invitation letters were posted at the beginning of each month during the inclusion period. The letters invited individuals to participate in the study 2 months after their 85^th^ birthday. In the case of no response to an invitation letter, a reminder was sent after 2 weeks. All responses were sent to the Department of Geriatric Medicine, Linköping University Hospital.

### Postal questionnaire

The postal questionnaire included questions on socio-demographics (housing, marital status, living situation, level of education, and previous occupation). Working status, measured by previous occupation, was classified into the following categories: low (blue collar), intermediate (white collar), and high (self-employed or academic profession) [18]. For non-participants, information about housing type was checked using the registered address.

The individuals were asked about their use of assistive technology (wheelchair, walker, crutch, vertically adjustable bed, bath/shower technology, adapted toilet, portable toilet, and gripper) and assistance needed (community assistance, transportation service, personal alarm, and food delivery). To evaluate the individual’s self-rated health, a visual analogue scale (VAS) was used ranging from 0 (worst imaginable health status) to 100 (best imaginable health status) [19]. Finally, the individuals provided information on the presence of chronic diseases.

### Morbidities and use of health services

The patients’ medical records are part of the electronic medical report system of the County Council of Östergötlands containing all health-care records (both inpatient and outpatient data) for all citizens of Linköping and the County of Östergötland. Older medical history was also checked in older paper medical records kept for all individuals at the central hospital archives of Linköping University Hospital. This procedure was performed by an experienced geriatrician. The research team compared the documentation in the medical records with the self-reported information in terms of diseases and drug treatments. The self-reported information was the response to two separate questions in the postal questionnaire, regarding chronic and acute medical conditions/diseases. All diseases/conditions indicated were noted for each patient. A disease or condition was only registered if there was clear documentation of the disease and its treatment, regardless of the patients’ self-reports. The 10^th^ version of the International Classification of Diseases (ICD-10) was used [20]. The presence of chronic disease was then registered if the disease fulfilled one or more of three criteria: the disease was permanently present, the disease was caused by an irreversible pathological condition, and treatment for the disease required rehabilitation or a long period of care. A predetermined list was made for disease categories: cardiovascular disease, cerebrovascular disease, respiratory disease, musculoskeletal disease, mental disease, neurological disease, digestive disorders, urological disorders, endocrine disease, hematological disorders, autoimmune disease, infection, skin changes and malignancy (solid and blood). In the present study, we chose a prevalence of more than 5% as the criterion for a common morbidity.

Data for the use of health services by each individual were provided by the local health care register. The records included visits to a GP, visits to an ER and hospitalization during 2007.

### Statistical analyses

The SPSS Statistical package (version 20.0) was used for the data analyses. The differences between men and women were assessed using the Student’s t-test for normally distributed continuous variables, Mann–Whitney U-test for non-Gaussian distributed variables, and Pearson Chi-square test for categorical variables. Effect size was calculated using Cohen’s d for Student’s t-test, rank-biserial correlation coefficient r (r_rb_) for the Mann–Whitney U-test and Cramér’s phi (**φ**_***c***_) for the Pearson Chi-square test.

A hierarchical cluster dendrogram was generated using Yule’s Q as the similarity measure between clusters, with a higher value indicating greater similarity measurement. Yule’s Q correlation matrix was calculated as a transformation of the odds ratio (OR) between two variables from (0 to infinity) to (-1 to +1): Q = (OR – 1) / (1 + OR) [21,22]. We chose the average linkage between groups for the agglomeration because this method takes into account the cluster structure and is relatively robust [23].

To determine predictors of an ER visit or hospitalization, logistic regression was performed with a forward stepwise method (using a likelihood ratio, with entrance/exit tolerances of 0.05/0.10). Model 1 used all single morbidities as candidate variables. Model 2 substituted cluster scores for single morbidities. Interaction of morbidity clusters was included in Model 3. According to John et al. [13], the effects of multimorbidity patterns are evaluated in the form of cluster scores (a count of all morbidities in one cluster) and their interactions (multiplication of two cluster scores, to determine if the clusters’ effects are dependent on each other). Other candidate variables such as socio-demographic factors, individuals’ needs, self-rated health, and the number of visits to a GP were included for model fitting [10]. Collinearity and correlation were analyzed before model fitting. Marital status and living situations were not included concurrently in the analysis owing to high collinearity (r > 0.6). The Nagelkerke R^2^ (Cox and Snell R^2^ adjusted, range 0–1) was used to estimate the amount of variance in the outcomes explained by the predictors [24].

### Ethical aspects

The local Ethical Committee approved the study (Dnr141-06, Linköping), and written informed consent was obtained from all participants and/or their relatives. All participants were informed that taking part in the project was voluntary and participation could be terminated at any moment.

## Results

### Completeness, representativeness, and sample characteristics

Twelve individuals died before completing the questionnaire and 52 (8%) individuals did not respond to the invitation letter even after the reminder. A total of 496 individuals (189 men and 307 women, 76%) completed the questionnaire. No gender difference was found between participants and non-participants (men vs. women, 46 vs. 108, χ^2^ = 3.452, df = 1, p = 0.063). A larger proportion of non-participants (45/154, 30%) than participants (55/496, 11%) lived in sheltered accommodation/nursing homes (χ^2^ = 29.679, df = 1, p < 0.001). Table 1 summarizes baseline characteristics of the participants. More women than men were living by themselves, had lower education, had lower working status, and used more assistive technology and assistance. Despite the statistical significance, the differences correspond to a small effect size. The most frequently used assistive technology—a walker—was related to improving mobility (40% of all participants; men vs. women, 23% vs. 52%). Food delivery was the only item of assistance reported by few elderly (men vs. women, 10/189 vs. 26/307, χ^2^ = 1.64, df = 1, p = 0.2). The elderly perceived themselves to be in general good health (score of self-rated health ≥ 60) and men were even more positive than women in this study. During the observation year, over three-quarters of the elderly (men vs. women, 138 vs. 242, χ^2^ = 2.205, df = 1, p = 0.138) had visited a GP, but less than one-third had visited an ER or been hospitalized. In absolute numbers, almost twice as many women as men visited an ER or had been hospitalized.

**Table 1:** Characteristics of the participants

**Characteristics**	**Men n = 189**	**Women n = 307**	***p*-value (statistic)**	**Effect size**
Type of housing, n (%)			0.079 (χ^2^ = 3.08, df = 1) ^a^	φ_c_ = 0.079
Ordinary housing	174 (92)	267 (87)		
Sheltered accommodation/Nursing home	15 (8)	40 (13)		
Marital status, n (%)			<0.001(χ^2^ = 56.78, df = 1) ^a^	φ_c_ = 0.34
Married/Cohabitated	142 (75)	124 (40)		
Widowed/Divorced/Unmarried	47 (25)	183 (60)		
Living situation, n (%)			<0.001 (χ^2^ = 61.17, df = 1) ^a^	φ_c_ = -0.35
Alone	68 (36)	220 (72)		
With others	121 (64)	87 (28)		
Level of education, n (%)			<0.001(χ^2^ = 6.57, df = 1) ^a^	φ_c_ = -0.18
≤ 7 years	97 (52)	188 (64)		
> 7 years	89 (48)	106 (36)		
Working status, n (%)			0.004 (χ^2^ = 10.83, df = 2) ^a^	φ_c_ = -0.15
Low (blue collar)	81(44)	174(59)		
Intermediate (white collar)	85(46)	103(35)		
High (self-employed or academic profession)	17(9)	16(6)		
Use of assistive technology, n (%)	80 (43)	212 (70)	<0.001(χ^2^ = 34.33, df = 1) ^a^	φ_c_ = 0.26
No. of used assistive technology, Median, (IQR)	0 (0–2)	2 (0–3)	<0.001(U = 20 116, df = 490) ^b^	r_rb_ = 0.26
Assistance needed, n (%)	75 (40)	209 (68)	<0.001 (χ^2^ = 37.11, df = 1) ^a^	φ_c_ = 0.28
No. of used assistance service, Median, (IQR)	0 (0–1)	1 (1–2)	<0.001(U = 19 001, df = 488) ^b^	r_rb_ = 0.3
Self-rated Health (range 0–100), Mean ± SD	69 ± 19	65 ± 20	0.018 (t = -2.37, df = 435) ^c^	Cohen’s d = 0.21
No. of GP visits, Median (IQR)	1 (0–3)	2 (1–3)	0.057 (U = 26 119, df = 494) ^b^	r_rb_ = 0.09
Any visit to ER, n (%)	55 (31)	95 (29)	0.664 (χ^2^ = 0.19, df = 1) ^a^	φ_c_ = 0.02
Any in-patient hospitalization, n (%)	44 (25)	79 (23)	0.539 (χ^2^ = 0.38, df = 1) ^a^	φ_c_ = 0.03

*GP*, General practitioner; *ER*, Emergency Room;

Number of subjects, % of subjects, means with standard deviations (SD), and median with interquartile range (IQR) of variables are shown.

^a^ Pearson Chi-square, ^b^ Mann–Whitney U Test; ^c^ Student’s t test; φ_c_*:* Cramér’s phi; r_rb_: rank-biserial correlation coefficient r.

Table 2 gives the rates of most common morbidities according to gender. The significant differences were the greater proportions of men with myocardial infarction and malignancy and the greater proportions of women suffering urinary incontinence, affective disease, dementia and osteoporosis.

**Table 2:** Prevalence of diagnosed chronic diseases (n = 496)

	**Total n (%)**	**Men n (%)**	**Women n (%)**	***p*-value (statistic)**
Hypertension	250 (50.4)	97 (51.3)	153 (49.8)	0.748 (χ^2^ = 0.10)
Hyperlipidemia	107 (21.6)	53 (28)	54 (17.6)	0.006 (χ^2^ = 7.56)
Urinary incontinence	103 (20.8)	19 (10.1)	84 (27.4)	<0.001 (χ^2^ = 21.3)
Arrhythmia	78 (15.7)	29 (15.3)	49 (16)	0.115 (χ^2^ = 0.03)
Heart failure	75 (15.1)	33 (17.5)	42 (13.7)	0.254 (χ^2^ = 1.30)
Diabetes	75 (15.1)	27 (14.3)	48 (15.6)	0.684 (χ^2^ = 0.17)
Stroke	58 (11.7)	23 (12.2)	35 (11.4)	0.796 (χ^2^ = 0.07)
Myocardial infarction	55 (11.1)	30 (15.9)	25 (8.1)	0.008 (χ^2^ = 7.09)
Affective diseases	60 (12.1)	14 (7.4)	46 (15)	0.012 (χ^2^ = 6.32)
Malignancy	50 (10.1)	28 (14.3)	22 (7.2)	0.006 (χ^2^ = 7.48)
Asthma or COPD	45 (9.1)	20 (10.6)	25 (8.1)	0.358 (χ^2^ = 0.84)
Osteoarthritis	39 (8.3)	11 (5.8)	28 (9.8)	0.185 (χ^2^ = 1.76)
Thrombosis or PVD	35 (7.1)	14 (7.9)	21 (6.5)	0.811 (χ^2^ = 0.06)
Dementia	33 (6.7)	7 (3.7)	26 (8.5)	0.039 (χ^2^ = 4.28)
Thyroid dysfunction	33 (6.7)	8 (4.2)	25 (8.1)	0.09 (χ^2^ = 2.88)
Osteoporosis	24 (4.8)	1 (0.5)	23 (7.5)	<0.001 (χ^2^ = 12.32)
Multimorbidity (≥2 chronic diseases)	339 (68.3)	134 (70.8)	205 (66.8)	0.338 (χ^2^ = 0.92)

*COPD*, Chronic Obstructive Disease; *PVD*, Periphery Vascular Disease.

Data were analyzed using Chi-square, df =1.

### Morbidity clusters

**Figure 1:**
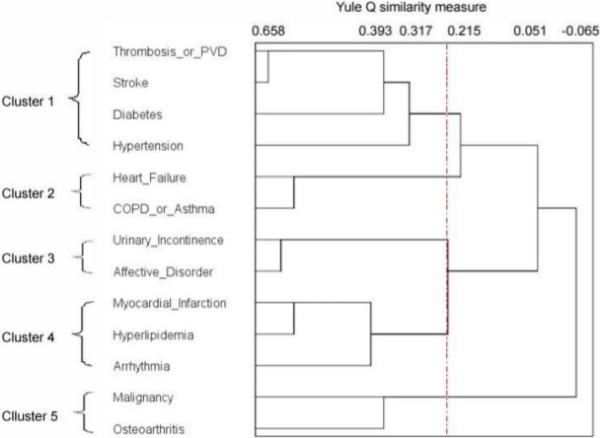
**Men’s morbidity clusters.** In the tree diagram, the distance between two clusters (or variables) is calculated according to the measure of similarity (Yule’s Q) and the cluster algorithm (average linkage between groups). The shorter the distance, the closer are the clusters. Three to five clusters are obtained by shifting the cut-off (vertical line) between Q values of 0.2 and 0.3. We evaluate that a five-cluster solution identifies most clinically meaningful multi-morbidity. The agglomerative coefficients given to the terminal node in each cluster are: Cluster 1, 0.317 (OR 1.9); Cluster 2, 0.587 (OR 3.8); Cluster 3, 0.62 (OR 4.3); Cluster 4, 0.581 (OR 3.8); Cluster 5, 0.393 (OR 2.3).

**Figure 2:**
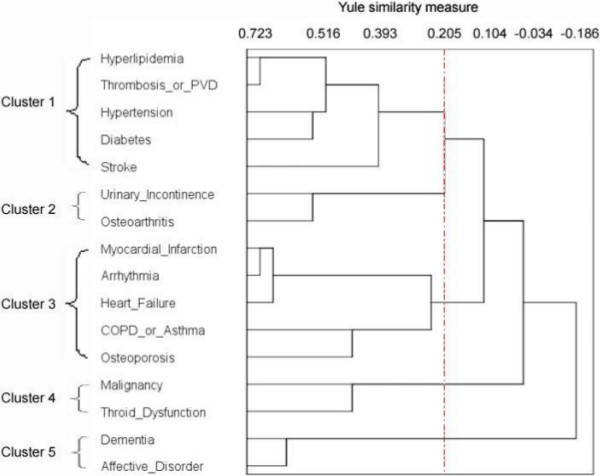
**Women’s morbidity clusters.** Four to six clusters are obtained by shifting the cut-off (vertical line) between Q values of 0.2 and 0.3. We evaluate that a five-cluster solution identifies most clinically meaningful multi-morbidity. The agglomerative coefficients given to the terminal node in each cluster are: Cluster 1, 0.393 (OR 2.3); Cluster 2, 0.557 (OR 3.5); Cluster 3, 0.244 (OR 1.6); Cluster 4, 0.45 (OR 2.6); Cluster 5, 0.619 (OR 4.3).

Using the measure of similarity (Yule’s Q) and the cluster algorithm (average linkage between groups), we found a large decline in agglomerative coefficients between 0.2 and 0.3, indicating an increase in heterogeneity between clusters. A cut-off in this range of coefficients provided three–five clusters for men (Figure 1) and four–six clusters for women (Figure 2). A higher cut-off coefficient resulted in several smaller clusters whereas a lower cut-off coefficient provided larger clusters. We evaluated that a five-cluster structure identifies most clinically meaningful multimorbidity for both genders. To show the magnitude of similarity between clusters/variables, we took Cluster 1 as an example and read off the distance for each node in Cluster 1 in the dendrograms.

In the men’s dendrogram, Cluster 1 was identified as a vascular cluster. Heart and pulmonary conditions were structured in Cluster 2 (cardiopulmonary) and Cluster 4 (cardiac). Two clusters were related to aging: Cluster 3 containing a somatic–mental combination and Cluster 5 aggregating malignancy with osteoarthritis.

In the women’s dendrogram, the vascular cluster (Cluster 1) was similar to that in the men’s dendrogram but included hyperlipidemia. The cardiopulmonary cluster (Cluster 3) was larger than that of men; myocardial infarction, arrhythmia, and heart failure were connected, and chronic obstructive pulmonary disease (COPD)/asthma was associated with osteoporosis. There were combinations related to aging in Cluster 2 where urinary incontinence was combined with osteoarthritis and in Cluster 4 where malignancy and thyroid dysfunction were merged. Finally, a mental disease cluster (Cluster 5) comprised dementia and affective disorders.

### Factors associated with an ER visit

As illustrated in Tables 3 and 4, single-morbidity models (Model 1) explained more variance than did morbidity-cluster models (Model 2). Heart failure was the most significant factor associated with ER visits for both men and women (Model 1). The men’s cardiac cluster (Cluster 4) and women’s cardiopulmonary cluster (Cluster 3) led to an increased likelihood of an ER visit (Model 2). Model 3 for cluster interaction was not constructed, because there was no significant cluster interaction.

**Table 3:** Association of single morbidity and morbidity clusters with ER visits in men

**Model 1 single morbidity**		**Model 2 morbidity clusters**	
Predictors	OR (95% CI); *p*	Predictors	OR (95% CI); *p*
Heart failure	2.4 (1–5.7); 0.043	Cluster 4	1.6 (1–2.5); 0.036
No. of GP visits	1.3 (1.1-1.5); 0.006	No. of GP visits	1.3 (1.1-1.5); 0.004
Nagelkerke R^2^	0.135	Nagelkerke R^2^	0.11

Odds Ratios (ORs) with 95% Confidence Intervals (CI) in parentheses and *p*-value are shown.

Cluster4: hyperlipidemia, myocardial infarction, and arrhythmia.

**Predictors excluded in model 1:** type of housing, marital status, level of education, working status, no. of used assistive tecknology, no. of used assistance service, self-rated health, thrombosis/PVD, stroke, diabetes, hypertension, COPD/asthma, urinary incontinence, affective disorder, myocardial infarction, hyperlipidemia, arrythmia, malignancy, and osteoarthritis.

**Predictors excluded in model 2:** type of housing, marital status, level of education, working status, no. of used assistive tecknology, no. of used assistance service, self-rated health, Cluster 1, Cluster 2, Cluster 3 and Cluster 5.

**Table 4:** Association of single morbidity and morbidity clusters with ER visits in women

**Model 1 single morbidity**		**Model 2 morbidity clusters**	
Predictors	OR (95% CI); *p*	Predictors	OR (95% CI); *p*
Low working status	reference	Cluster 3	1.5 (1.1-2); 0.021
Middle working status	2.2 (1.1-4.1); 0.018	No. of GP visits	1.4 (1.2-1.6); <0.001
High working status	3.5 (1.1-11.3); 0.036		
Heart failure	3 (1.3-6.9); 0.01		
Arrhythmia	2.2 (1–4.8); 0.05		
Diabetes	0.3 (0.1-0.9); 0.027		
No. of GP visits	1.3 (1.1-1.6); <0.001		
Nagelkerke R^2^	0.219	Nagelkerke R^2^	0.143

Odds Ratios (ORs) with 95% Confidence Intervals (CI) in parentheses and *p*-value are shown.

GP: General Practioner;

Cluster3: myocardial infarction, arrhythmia, heart failure, COPD/asthma, and osteoporosis.

**Predictors excluded in model 1:** type of housing, marital status, level of education, no. of used assistive tecknology, no. of used assistance service, self-rated health, hyperlipidemia, thrombosis/PVD, hypertension, stroke, urinary incontinence, osteoarthritis, myocardial infarction, COPD/asthma, osteoporosis, malignancy, thyroid dysfunction, dementia, and affective disorder.

**Predictors excluded in model 2:** type of housing, marital status, level of education, working status, no. of used assistive tecknology, no. of used assistance service, self-rated health, Cluster 1, Cluster 2, Cluster 4, and Cluster 5.

### Factors associated with hospitalization

Morbidity clustering (Model 2) and cluster interactions (Model 3) explained more variance than the single-morbidity model (Model 1) (Tables 5 and 6).

**Table 5:** Association of single morbidity, morbidity clusters, and cluster interactions with hospitalization in men

**Model1 single morbidity**		**Model 2 morbidity clusters**		**Model 3 interactions between morbidity clusters**	
Predictors	OR (95% CI); *p*	Predictors	OR (95% CI); *p*	Predictors	OR (95% CI); *p*
No. of used assistive technology	1.6 (1.2-2); <0.001	No. of used assistive technology	1.6 (1.3-2); <0.001	No. of used assistive technology	1.6 (1.2-2); <0.001
No. of GP visits	1.2 (1.0-1.5); 0.028	No. of GP visits	1.2 (1.0-1.5); 0.032	No. of GP visits	1.2 (1.0-1.5); 0.049
		Cluster 4	1.6 (1.0-2.7); 0.048	Cluster 2* Cluster 4	1.6 (1.0-2.4); 0.042
Nagelkerke R^2^	0.188	Nagelkerke R^2^	0.219	Nagelkerke R^2^	0.22

Odds Ratios (ORs) with 95% Confidence Intervals (CI) in parentheses and *p*-values are shown.

*GP*, General practitioner;

Cluster 2: heart failure, asthma/COPD; Cluster 4: hyperlipidemia, myocardial infarction, and arrhythmia;

**Predictors excluded in model 1:** type of housing, marital status, level of education, working status, no. of used assistance service, self-rated health, thrombosis/PVD, stroke, diabetes, hypertension, heart failure, COPD/asthma, urinary incontinence, affective disorder, myocardial infarction, hyperlipidemia, arrythmia, malignancy, and osteoarthritis.

**Predictors excluded in model 2:** type of housing, marital status, level of education, working status, no. of used assistance service, self-rated health, Cluster 1, Cluster 2, Cluster 3, and Cluster 5.

**Predictors excluded in model 3:** type of housing, marital status, level of education, working status, no. of used assistance service, self-rated health, Cluster 1, Cluster 2, Cluster 3, Cluster 4, Cluster 5, Cluster 1*Cluster 4, Cluster 3*Cluster 4, Cluster 5*Cluster 4.

**Table 6:** Association of single morbidity, morbidity clusters, and cluster interactions with hospitalization in women

**Model 1 single morbidity**		**Model 2 morbidity clusters**		**Model 3 Iinteractions between morbidity clusters**	
Predictors	OR (95% CI); *p*	Predictors	OR (95% CI); *p*	Predictors	OR (95% CI); *p*
No. of GP visits	1.4 (1.2-1.6); <0.001	No. of GP visits	1.3 (1.1-1.6); <0.001	Sheltered accommodation/ Nursing home	2.5 (1.0-5.9); 0.044
Heart failure	3.4 (1.6-7.3); 0.002	Cluster 2	0.4 (0.2-0.8); 0.006	No. of GP visits	1.4 (1.2-1.6); <0.001
Urinary incontinence	0.4 (0.2-0.8); 0.012	Cluster 3	1.7 (1.2-2.4); 0.004	Cluster 3	2.3 (1.5-3.5); <0.001
				Cluster 2* Cluster 3	0.5 (0.3-0.8); 0.005
Nagelkerke R^2^	0.19	Nagelkerke R^2^	0.193	Nagelkerke R^2^	0.213

Odds Ratios (ORs) with 95% Confidence Intervals (CI) in parentheses and *p*-values are shown.

*GP*, General practitioner;

Cluster 2: incontinence, osteoarthritis; Cluster 3: myocardial infarction, arrhythmia, heart failure, asthma/COPD, and osteoporosis.

**Predictors excluded in model 1:** type of housing, marital status, level of education, working status, no. of used assistive tecknology, no. of used assistance service, self-rated health, malignancy, hypertension, myocardial infarction, arrythmia, hyperlipidemia, COPD/asthma, diabetes, dementia, affective disorder, thyroid dysfunction, osteoporosis, osteoarthritis, thrombosis/PVD, and stroke.

**Predictors excluded in model 2:** type of housing, marital status, level of education, working status, no. of used assistive tecknology, no. of used assistance service, self-rated health, Cluster 1, Cluster 4, and Cluster 5.

**Predictors excluded in model 3:** marital status, level of education, working status, no. of used assistive tecknology, no. of used assistance service, self-rated health, Cluster 1, Cluster 2, Cluster 4, Cluster 5, Cluster 1*Cluster 2, Cluster 4* Cluster 2, Cluster 5* Cluster 2, Cluster 1* Cluster 3, Cluster 4*Cluster 3, Cluster 5*Cluster 3.

No single morbidity was significantly related to men’s hospitalization. The cardiac cluster (Cluster 4) and its combination with the cardiopulmonary cluster (Cluster 2) were significant with respect to hospitalization. The variance had an overall increase of 3.2% from Model 1 to Model 3.

For women, heart failure was positively associated with hospitalization and urinary incontinence had an inverse association. The clusters (Cluster 2 and 3) including these two morbidities appeared in Model 2. In Model 3, the cardiopulmonary cluster (Cluster 3) had a stronger effect than that in Model 2. However, Cluster 2 dampened the effect via a cluster interaction with Cluster 3.

## Discussion

Many very old people inevitably need daily assistance and health service as a result of functional impairment and illness. The existence of multimorbidity has a complex effect on the use of health services. Unfortunately, the complexity and conjoint effects are often overlooked. In the present study, rather than just focusing on a single diagnosis, we studied multimorbidity patterns in relation to the use of health services. Our major findings were that patterns of cardiac and pulmonary conditions were better associated than any single morbidity with hospitalization and that heart failure as a single morbidity was better associated than multimorbidity patterns with ER visits. Gender stratification simplified the comprehensive role played by gender in morbidity prevalence and related factors associated with the use of health services.

### Morbidity clusters

Beyond the statistical results from cluster analysis, some patterns of multimorbidity are expected and supported with findings from other studies. First, in Cluster 1, all morbidities shared the common pathophysiological mechanism of vascular disorders except diabetes. However, we still have good reason to believe that in the long run very old people who have complications associated with diabetes have other co-occurring vascular morbidities. Similar findings were also reported by previous studies using cluster analysis [13,15]. Second, the cardiopulmonary cluster is another expected cluster. Heart failure in the men’s cardiopulmonary cluster was only related to COPD/asthma. The cluster was closer to vascular diseases (Cluster 1) than the cardiac cluster (Cluster 4). The women’s cardiopulmonary cluster contained all heart conditions as well as COPD/asthma. COPD/asthma was first linked to osteoporosis, suggesting osteoporosis was a consequence of long-term treatment of corticosteroids for COPD/asthma patients [25,26]. This cardiopulmonary pattern was also reported by Marengoni et al. [14] and John et al. [13] but with no gender specificity. A third finding is the clusters of mental diseases. The women’s mental and somatic morbidities were independent of each other. Comparatively, men had a somatic–mental cluster as only affective disorder was included in the analysis. Its association with urinary incontinence was not formally documented in any psychiatric journal according to Vasudev et al. [27] even though the impact of urinary incontinence on mental health has been reported by other researchers [28,29].

Some morbidities emerged in the same cluster but did not seem to follow pathophysiological pathways such as urinary incontinence and osteoarthritis (Cluster 2 in women). In women, osteoarthritis-related disability may negatively affect urinary control [30]. Another exception is the comorbidity of malignancy. It is difficult to anticipate which comorbidity coexists with a certain type of malignancy, since cancer patients manifest multiple health problems [31]. One reflection from daily clinical practice is that patients with a malignancy diagnosis usually have received complete clinical and laboratory examinations, and therefore, some comorbidity such as osteoarthritis and thyroid dysfunction would not be missed. Another hypothesis is based on the selection of survivals of concurrent ailments. Among cancer patients, some co-occurrences (e.g., severe heart disease) are more likely than others (e.g., osteoarthritis) to cause a high risk of mortality.

### Multimorbidity patterns associated with ER visits and hospitalization

Patients using ER services are heterogeneous with respect to the medical services they require. The slightly lower R^2^ in the morbidity cluster models reveals that the selected morbidity cluster (men’s cardiac cluster and women’s cardiopulmonary cluster) did not improve explained variance. In other words, single-morbidity models are more precise in predicting ER visits. A reflection of real clinical practice is that a single morbidity (e.g., heart failure) as a medical condition may already be enough for an ER visit. Unexpectedly, several common morbidities such as COPD/asthma, stroke and even myocardial infarct were not significantly related to ER visits in this study population. Seemingly, in this very old population, these diagnoses were not clearly related to exacerbations or new attacks, but more possibly suggested permanent chronic conditions in patients’ medical records.

In terms of hospitalization, our results are consistent with those of other studies that multimorbid patients were more likely to be hospitalized [32,33]. The advantage of our approach is that morbidity cluster and cluster interaction models provide more information. Unlike the counts of morbidities, where all morbidities are equally scored irrespective of their inner relationship, morbidity cluster and cluster interaction models address what morbidity cluster was the leading cause of hospitalization. For both men and women, the cardiac and pulmonary condition was a major factor associated with hospitalization. For women, urinary incontinence and its comorbidity with osteoarthritis suggests that old women with certain conditions might be treated using care services other than hospitalization (e.g., primary care).

### Methodological issues

There is no consensus about how to best measure multimorbidity. According to the theory that the associations among morbidities must be involved when comorbidity rates exceed those that are statistically expected (coincidental) [34], hierarchical cluster analysis helps identify the conjunction between morbidities in a small population with a high prevalence of multimorbidity. Cluster score and cluster interactions have revealed synergistic effects on associative morbidities [13]. However, we realize that very different results may be obtained from the same data using different hierarchical clustering methods [23]. It is of great importance to relate the statistical results to real-life clinical practice so as to verify the interpretable clusters.

In the logistic regression models, the low R^2^ reminds us that reasons for the use of health services are multifaceted phenomena. According to Andersens’ behavioral model, the use of health services is determined by predisposing characteristics (e.g., demographics, social structure, and health belief), enabling resources (e.g., the number of medical personnel and facilities), or a need for health care (health conditions including mortality, morbidity, and disability) [35]. Even if need is a dominant reason why older people use the ER [36,37], the measures of need as well as other contextual factors can vary [38]. In the present study, an increased number of GP visits reflected the medical needs of very old peoples. Greater use of assistive technology by older men provides information about their severe physical disability or illness because men are more reluctant than women to use assistive technology that brings them shame, embarrassment, and feelings of victimization [39]. Working status and education were measured separately instead of transforming to socio-economic status. The effect of socio-economic status on the use of health service is not consistent in all studies [37,40,41], probably owing to the use of different measures and the different financing of health care.

### Limitations

A number of limitations of the present study should be mentioned. First, some morbidities (e.g., arthritis, anemia, and hip fracture) that were not included in the analysis had higher prevalence in other studies [13-15,42]. We cannot draw any gender-specific conclusion in the present study. Heterogeneity among populations needs to be considered. Second, diseases with no treatment and asymptomatic conditions could be missed by self-reported surveys and neglected by doctors when recording a medical history; e.g., anemia and osteoporosis. In particular, among non-participants having a high frequency of living in nursing homes, the extreme underestimation of dementia results in health services not being provided to individuals with cognitive impairment. Third, the financing and organization of health care in Sweden limits the generalizations of the findings as other countries may have different social or health care policy. Different welfare regimes affect the priorities of public resources and address inequality issues relating to the use of health services. Individuals with supplemental private health insurance may use private health services, but in this age group, the consumption of private health care is not common practice and it is not the focus of this paper.

## Conclusions

We identified a vascular cluster, cardiopulmonary cluster and clusters related to aging for a population of 85-year-old Swedish men and women. A cardiac cluster and somatic–mental cluster were found in the men’s cluster structure and a mental disease cluster in the women’s. We further explored these clusters in relation to hospitalization and ER visits. Patterns of cardiac and pulmonary conditions explained hospitalization better than any single morbidity, while heart failure as a single morbidity was superior to multimorbidity patterns in explaining ER visits.

At a population level, identifying what type of morbidity cluster exists may facilitate the capture of potential hospital users. A holistic approach to examining the patterns of multimorbidity and their relationship to the use of health services in a given population will be useful for planning local health care, allocating and prioritizing resources, and geriatric practice.

## Competing interest

The authors declared that they have no competing interest.

## Authors’ contributions

DHJ: retrieval of literature, analysis (design and performance) and interpretation of data, drafting of manuscript. WE: study concept and design, acquisition of subjects and data, manuscript development. MJ: study concept and design, data interpretation, and writing the final manuscript from the first draft. All authors read the manuscript and approved it for publication.

## Pre-publication history

The pre-publication history for this paper can be accessed here:

http://www.biomedcentral.com/1471-2318/13/120/prepub
